# On the Surgical Treatment of Polypi of the Larynx, and Oedema of the Glottis

**Published:** 1852-09

**Authors:** 


ART. IV.—*On the Surgical Treatment of Polypi of the Larynx, and Oedema of the Glottis.* By HORACE GREEN, A. M., M. D., President of the Faculty, and Professor of the Theory and Practice of Medicine, in the New York Medical College; Member of the American Medical Association; Fellow of the College of Physicians and Surgeons, N. Y.; and Honorary Member of the Philadelphia Medical Society. New York: G. P. Putnam: 1852: pp. 121: 8vo.

The affections of which this volume treats are of so rare occurrence, that they have attracted comparatively but little attention, but they are of so fatal a character, that they deserve to be carefully investigated. Dr. Green believes that foreign growths in the opening of the air-passages have often been overlooked where they were the cause of death, and that when the attention of the profession comes to be directed to this subject, it will be found that the existence of Polypus and other excrescences in these passages, "is an occurrence taking place much more frequently than has been supposed by medical practitioners." He has collected together the well authenticated histories of this disease, which amount to only about forty cases, of which four, or one-tenth, have occurred in his own practice within the past five or six years. Two others in the same time have been observed by Professor Parker, of New York; and we think with the author, that amongst physicians of long experience and extensive opportunities for observation, many on reading the histories of the cases here given will recall the occurrence of similar cases in their own practice.

Of all the cases referred to by Prof. Green, the result in every instance, except three, was fatal, the life of one having been saved by laryngo-tracheotomy, performed by a German surgeon, and the other two fortunate cases having been treated by our author.

The diagnosis of polypus of the larynx is obscure. Ehrmann asserts that there is but one certain sign—the discharge, by expectoration, of some portions of the tumor, as occurred in the case for which he performed the successful operation. In some cases the tumor may be seen, but neither of these evidences are afforded until the disease has advanced to a very formidable point, and in fact the patients often die before any such indications appear. Sometimes the tumors are sessile, but in most cases, they are pediculated, and then the patients may perceive their movements during respiration.

"An alteration in the sound of the voice is one of the most constant symptoms in polypus of the larynx."

This alteration may be slight, or it may amount to complete aphonia, it may occur suddenly, or may come on gradually. Dyspnea is another prominent symptom, slight, it may be, at first, but augmenting with the progress of the disease until it threatens suffocation.

The only remedy for this affection is the removal of the tumor, to detect which the patient, in cases where one is suspected, is to be placed opposite a good light, with his head properly supported; when the operator depresses the tongue with a spatula and drags it forward until the epiglottis is brought well into view. "At this moment the patient should be directed to cough forcibly, when a portion of the polypus, if it be floating in the cavity of the glottis, as is frequently the case, may be seen, momentarily, over the aryteno-epiglottic border." If this should fail, an attempt must be made to feel it with the finger. Its existence once detected, the operation is thus performed: The patient is placed as before described for examination, when the tumor, if seen, is caught with the double hook, held in the left hand of the operator. If successful in this, with the probe-pointed knife in the right hand, he is to divide the pedicle of the polypus, and with the tenaculum remove it from the larynx. In case no tumor can be detected upon repeated examinations, Dr. Green recommends the application of a strong solution of nitrate of silver to the glottis, and into the larynx of the patient; and should success attend neither plan, then he urges laryngotomy as the last resort.

We must notice briefly Dr. Green's treatment of *œdema glottidis*, the fatality of which disease may be inferred from the fact, that Dr. Bayle records but one case of recovery out of seventeen, Valleix but nine out of forty, and Sestier but forty-one out of one hundred and sixty-eight cases. His medication is topical. He applies a strong solution of nitrate of silver, sixty grains to the ounce of water, to the epiglottis and lips of the glottis, and by means of a sponge, passes some of the fluid into the ventricles of the larynx. He remarks that inflammation of the larynx, of whatever grade, is aggravated by the application of a weak preparation of the nitrate. A concentrated solution, on the contrary, arrests or changes the diseased action. He adds:

"The first application with the sponge-probang should be made to the pharynx, and the top of the epiglottis; and after a delay of ten or fifteen minutes, the measure may be repeated, and the sponge, wet with the solution, be freely applied to the base of the epiglottis, and over the edematous lips of the glottis. This application should be repeated every hour, or two hours, according to the urgency of the disease, and the effect produced by the operation, and an attempt should be made each time to carry the sponge between the lips of the glottis. As the edema at the opening of the larynx subsides, this may be done, and the application of the caustic solution, be made to the interior of the glottis."

We are sure that we need not urge our readers to make themselves familiar with the contents of this volume—in our judgment one of the most valuable contribulions to practical medicine yet made in our country.

